# On the Nerves of the Gravid Uterus

**Published:** 1844-03-23

**Authors:** R. D. Grainger

**Affiliations:** Lecturer on Anatomy at St. Thomas’s Hospital


### ON THE NERVES OF THE GRAVID UTERUS.

BY R. D. GRAINGER, ESQ.,

Lecturer on Anatomy at St. Thomas's Hospital.

I have derived great pleasure from examining the dissections of the uterine, vaginal, and vesical nerves, made by Dr. Lee. The trunk and branches of the sympathetic nerve being left, as well as the trunks of some of the sacral nerves, a satisfactory clue is afforded in the examination. The injections of the blood-vessels render a further and valuable aid in testing what are, and what are not nervous fibrils.

After carefully inspecting and examining these beautiful dissections, I have no hesitation in expressing my conviction that they bear out, fully and entirely, the delineations and descriptions published by Dr. Lee.

1. The preparations show an unequivocal continuity of fibres proceeding from undisputed nervous structures, the sympathetic and sacral nerves, to the newly discovered ganglia of the uterus, vagina, and ureter.

2. The nervous branches of the newly discovered ganglia join, in various directions, with acknowledged nerves, such as those of the inferior mesenteric plexus, furnishing the hæmorrhoidal nerves, and, with the spermatic nerves, descending on the uterus, from the folds of the broad ligaments.

3. The occurrence of small ganglia and gangliform enlargements on the newly discovered nerves, are very characteristic, and corroborative of their real nature.

4. The nerves are accompanied by injected blood-vessels in a manner that is not seen in elastic tissue, although usual with the ganglionic nerves.

5. The ganglia discovered by Dr. Lee present in their form and disposition, and in the openings which they possess, a perfect and entire correspondence with the larger ganglia of the sympathetic.

After the examination which I have made, it certainly appears to be impossible for any one to arrive at a just conclusion respecting the true character of Dr. Lee's description without a careful inspection of his preparations. It is proper to add that I have not yet had an opportunity of making a microscopical examination.—*Lon. Lancet*, Dec. 9, 1843.

As an instance of the love for manual operations may be cited a case occurring not long ago in a French Hospital. A woman in this establishment had arrived at her full term of gestation, and the pelvis appeared too narrow to admit of the egress of the child. The surgeon in charge of the patient proposed the Cæsarian operation, but a majority of his colleagues determined to postpone this for a period. Determined not to be baulked, however, the surgeon resolved to perform the operation; though, fearing to fly alone in the face of the previous consultation, he went to secure the presence and co-operation of a distinguished obstetric practitioner. Both soon arrived, bistoury in hand, at the bedside of the unfortunate patient. But nature had got the start of art. While the deliberations had been going on, so had labour, the result being a fine boy, who had preferred coming into the world by the old road.

*Ibid.*

